# Sleep Deprivation Exacerbates Ischemic Stroke Outcomes via *Akkermansia* Depletion and Metabolic Dysregulation

**DOI:** 10.1002/cns.70933

**Published:** 2026-05-20

**Authors:** Xin Nie, Sheng‐Yang Zhou, Le Yao, Lu‐Lu Tan, Xiao‐Yu Ma, Yi‐Meng Xia, Chun Cui, Wei‐Jiang Zhao, Chen‐Meng Qiao, Yan‐Qin Shen, Chao‐Sheng Li

**Affiliations:** ^1^ Department of Neurology Affiliated Hospital of Jiangnan University Wuxi Jiangsu China; ^2^ Lab of Neurodegeneration and Injury, Wuxi School of Medicine Jiangnan University Wuxi Jiangsu China; ^3^ MOE Medical Basic Research Innovation Center for Gut Microbiota and Chronic Diseases, School of Medicine Jiangnan University Wuxi Jiangsu China

**Keywords:** *Akkermansia*, gut microbiota, metabolite, middle cerebral artery occlusion, sleep deprivation

## Abstract

**Aims:**

Sleep disturbances are a potential risk factor for stroke. Our clinical cohort shows that insomnia is associated with a higher 90‐day stroke recurrence rate, prompting an investigation into the underlying mechanisms, particularly the role of the gut microbiota.

**Methods:**

We utilized a rat MCAO model of sleep deprivation (SD) combined with antibiotic (ABX)‐induced gut dysbiosis. Neurological deficits were assessed through behavioral tests, and cerebral infarct volume was measured using TTC staining. Structural changes in brain and intestinal tissues were observed by H&E staining, while the expression of blood–brain barrier (BBB)‐related proteins was detected by immunofluorescence and Western blot. Gut microbial composition was analyzed by 16S rRNA sequencing, and metabolomic analysis was performed using an untargeted LC–MS approach.

**Results:**

In rats with an intact microbiota, SD had a limited effect on MCAO‐induced ischemic injury. Strikingly, in ABX‐induced dysbiosis, SD‐exposed rats subjected to MCAO displayed more severe motor deficits, larger infarct volumes, heightened inflammation, and decreased expression of key intestinal and BBB proteins. Microbiota analysis identified *Akkermansia* as a potential key mediator, whose abundance was significantly decreased in the ABX + SD + MCAO group and negatively correlated with infarct size. Subsequent metabolomics profiling revealed a distinct dysregulation of *Akkermansia*‐associated metabolites (including PG(14:0/15:0), 3‐Methylbutylamine, N‐Acetylcytidine, and N‐Methylisoleucine).

**Conclusion:**

In conclusion, our study proposes a mechanism whereby SD worsens stroke outcomes by depleting *Akkermansia* and disrupting metabolic homeostasis.

## Graphical abstract

Under ABX‐induced gut dysbiosis, sleep deprivation reduces beneficial taxa and enriches pro‐inflammatory bacteria, leading to increased intestinal permeability and amplified peripheral inflammation, thereby exacerbating BBB disruption and neuroinflammation and worsening ischemic brain injury. Meanwhile, SD‐induced microbial alterations are accompanied by metabolic dysregulation, which is associated with poorer neurological recovery.

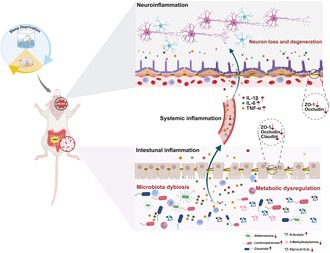

## 1 Introduction

Acute ischemic stroke (AIS) is a major cause of death and disability globally [1, 2]. Its complex pathophysiology severely limits the development and application of effective treatment strategies. The onset of this disease is influenced by multiple risk factors, including hypertension, diabetes, and sleep disorders [2, 3]. Previous studies have demonstrated that sleep disorders were a critical and independent risk factor for stroke [4, 5]. Large cohort studies and meta‐analyses have shown that insomnia and poor sleep quality significantly increase the risk of incident stroke, independent of conventional vascular risk factors [3, 6, 7]. Sleep‐disordered breathing, particularly obstructive sleep apnea, affects approximately 50%–70% of stroke patients and is linked to higher mortality and poorer recovery [8]. Moreover, accumulating evidence have suggested that the timing of sleep loss relative to ischemia is an important risk factor for outcomes [9]. Prolonged sleep deprivation (SD) before middle cerebral artery occlusion (MCAO) exacerbates infarct volume, neuroinflammation, and neurological deficits. In contrast, short‐term pre‐ischemic deprivation has been shown to induce paradoxical preconditioning‐like neuroprotection, reducing infarct size and improving survival [9, 10]. The proposed mechanisms underlying these effects include inflammation, blood–brain barrier (BBB) disruption, hypothalamic–pituitary–adrenal axis activation, and gut‐brain interactions [11, 12, 13, 14]. Notably, the brain is not an isolated organ; extensive crosstalk between the central nervous system and peripheral organs, particularly through the gut‐brain axis, critically influences stroke progression and recovery [15]. Blood‐derived factors act as key mediators of this central‐peripheral communication, transmitting peripheral signals that modulate neuroinflammation, BBB integrity, and neuronal survival. This framework provides a mechanistic basis for understanding how systemic perturbations, such as SD and gut dysbiosis, may converge to aggravate ischemic injury. Recent advances have revealed that the therapeutic window for ischemic stroke extends beyond the conventional time‐based paradigm to a molecular‐level framework, in which distinct pathophysiological cascades—including excitotoxicity, oxidative stress, inflammation, and BBB disruption—offer stage‐specific molecular targets for intervention [16]. This evolving understanding underscores the need to identify modifiable factors that exacerbate these molecular cascades. Nevertheless, these divergent findings indicate that pre‐stroke SD is a biologically relevant condition that significantly influences ischemic outcomes, although its precise role in modulating ischemic injury remains poorly understood.

Recent mechanistic studies have begun to illuminate how SD exerts its detrimental effects at the molecular level. For instance, chronic sleep fragmentation has been shown to induce metabolic imbalances including impaired glucose homeostasis and insulin sensitivity, with hypothalamic astrocyte‐derived acetate identified as a key protective metabolite that restores glycolysis and tricarboxylic acid cycle function [17]. These findings suggest that metabolic dysregulation represents a critical mechanistic node linking sleep disturbances to disease outcomes. Because the gut microbiota plays a central role in regulating host metabolism, it may represent an important mechanistic link between SD‐induced metabolic disturbance and stroke pathology. In recent years, increasing attention has been directed toward the role of the gut microbiota in stroke, given its emerging importance in disease onset, progression, and recovery [18, 19, 20]. Alpha diversity declines after stroke, and gut microbial dysbiosis amplifies neuropathological damage by linking metabolite toxicity, innate immune sensing, adaptive immune skewing, and barrier failure into a single cascade [21]. Dysbiotic consortia convert dietary choline to Trimethylamine N‐oxide, which primes platelets, promotes thrombosis, and propagates neuroinflammation [22]. The ensuing translocation of microbial products and cytokines sustains systemic inflammation, recruits primed lymphocytes to the ischemic brain, hyperactivates microglia, and aggravates neuronal injury [18]. Collectively, these microbiota‐host positive feedback loops aggravate stroke pathogenesis and shape clinical outcomes [23].

Growing evidence have confirmed separate associations between gut microbiota and stroke, and between SD and stroke [9, 24]. However, it remains unclear whether SD affects the pathological process of AIS by altering specific gut microbial members. In this context, the present study employed an antibiotic cocktail (ABX)‐treated MCAO model to investigate the relationship between SD and the gut microbiota in stroke.

## 2 Materials and Methods

### 2.1 Clinical Study Design and Participants

This multicenter retrospective cohort study included patients with AIS who underwent mechanical thrombectomy between January 2020 and December 2023 at three tertiary hospitals in Wuxi, China. Eligible patients were aged ≥ 18 years, presented with clinical symptoms consistent with AIS, had neuroimaging‐confirmed large‐vessel occlusion, and received MT within 24 h of symptom onset with successful reperfusion. Patients were excluded if they had intracranial hemorrhage, malignancy, epilepsy, or autoimmune disease; documented coagulopathy or thrombocytopenia; unsuccessful recanalization; fever or infection within 2 weeks prior to admission; or incomplete follow‐up data. The study was approved by the Ethics Committee of the Affiliated Hospital of Jiangnan University and conducted in accordance with the Declaration of Helsinki. A total of 404 AIS patients undergoing MT were screened across the three centers. After excluding 104 patients due to incomplete data, 300 were included in the final cohort. Among them, 114 patients met criteria for insomnia and 186 were classified as non‐insomnia. Baseline clinical characteristics—including demographics, vascular risk factors, ASPECTS score, procedural time metrics (puncture‐to‐reperfusion and onset‐to‐reperfusion times), intravenous thrombolysis status, reperfusion success, and hemorrhagic complications—were collected for comparison between groups.

All patients completed the Athens Insomnia Scale assessment [25]. Patients with an Athens Insomnia Scale score > 6 were categorized into the insomnia group, whereas those with a score ≤ 6 were categorized into the non‐insomnia group [25, 26]. Clinical outcomes were compared between the two groups. All patients were followed up by neurologists trained in standardized evaluation procedures via telephone, during which sleep status and 90‐day functional outcomes were documented.

### 2.2 Animal Experiments

Male Sprague–Dawley rats (5 weeks old) were obtained from SPF (Beijing) Biotechnology Co. Ltd. All animals were maintained in the Laboratory Animal Center of Jiangnan University under standard conditions (a 12/12‐h light/dark cycle, 24°C ± 2°C, and relative humidity of 55% ± 10%). Rats had ad libitum access to food and water throughout the study. After a 7‐day acclimatization period, rats were transferred to a specific pathogen‐free environment. Body weight was monitored daily during the experimental period. All experimental protocols were approved by the Animal Ethics Committee of Jiangnan University (Approval No. JN. No. 20240315S0900515).

Animal inclusion and exclusion criteria, mortality, and final sample allocation were defined as follows: Following a one‐week acclimatization period, 110 rats were initially included in the study. Of these, 11 died during or after surgery (Group III (NSD + MCAO): 3, Group IV (SD + MCAO): 2, Group V (ABX + NSD + MCAO): 3, Group VI (ABX + SD + MCAO): 3). Nine rats were excluded due to unsuccessful MCAO (Longa score = 0) or subarachnoid hemorrhage. The final sample sizes were 15 in each group. The SD protocol consisted of 18 h of deprivation per day for 14 consecutive days. Group I (NSD + Sham): Intragastric saline was administered under non‐sleep deprivation (NSD) conditions, followed by sham surgery. Group II (SD + Sham): Intragastric saline was administered during SD, followed by sham surgery. Group III (NSD + MCAO): Intragastric saline was administered under NSD conditions, followed by MCAO. Group IV (SD + MCAO): Intragastric saline was administered during SD, followed by MCAO. Group V (ABX + NSD + MCAO): Intragastric ABX was administered under NSD conditions, followed by MCAO. Group VI (ABX + SD + MCAO): Intragastric ABX was administered during SD, followed by MCAO. For behavioral assessments (OFT, Longa, mNSS) and body weight monitoring, all surviving animals were included (*n* = 15 per group). For histological analyses (HE staining, histological scoring), a subset of animals was randomly selected (*n* = 4 per group). For Western blot and immunofluorescence analyses, *n* = 4 per group were used. For TTC staining, *n* = 3 per group were used. For ELISA, RT‐qPCR, 16S rRNA sequencing, and metabolite measurements, *n* = 6 per group were used.

Following the final behavioral assessments, all rats were euthanized for collection of brain, colon, and fecal samples for molecular analysis. A schematic representation of the experimental design and timeline is shown below (Figure S1).

Antibiotic Treatment: In the ABX‐treated groups (ABX + NSD + MCAO and ABX + SD + MCAO), rats were administered a broad‐spectrum ABX via oral gavage once daily from day 0 to day 7 to deplete their gut microbiota. This cocktail contained neomycin (100 mg/kg, HY‐B0470, MedChemExpress, USA), metronidazole (100 mg/kg, HY‐B0318, MedChemExpress, USA), ampicillin (100 mg/kg, HY‐B0522, MedChemExpress, USA), and vancomycin (50 mg/kg, V105495, Aladdin, China), as described previously [27]. Rats in the non‐ABX groups received an equal volume of 10 mL/kg sterile saline via oral gavage over the same period for comparison. The efficacy of ABX treatment in depleting gut microbiota was evaluated by 16S rRNA sequencing.

Sleep Deprivation Modeling: SD was induced using an adapted version of the modified multiple platform method, as described previously [28]. From day 8 to day 21, animals were subjected to SD for 18 h per day. Briefly, rats were placed on narrow circular platforms (6.5 cm in diameter) surrounded by water. This approach takes advantage of the muscle atonia that characterizes rapid eye movement (REM) sleep: upon entering REM phase, the animals lose muscle tone and fall into the water, thereby interrupting REM sleep. Control animals were maintained under identical conditions but placed on wider platforms that allowed normal sleep. Throughout the experimental period, all animals had ad libitum access to food and water. This method enables selective REM sleep disruption while maintaining comparable environmental conditions between experimental and control groups.

Middle Cerebral Artery Occlusion (MCAO) Model: Brain ischemia–reperfusion injury was induced by MCAO followed by reperfusion, as previously described [29]. Briefly, rats were anesthetized with 4% isoflurane for induction and maintained under 2% isoflurane. Throughout the surgery, core body temperature was maintained at 37.0°C ± 0.5°C using a temperature‐controlled heating pad. A midline cervical incision was made to expose the left carotid artery. The left external carotid artery (ECA), left internal carotid artery (ICA), and left common carotid artery (CCA) were carefully dissected free from the surrounding connective tissue and the vagus nerve. The proximal portions of the left ECA and left CCA were ligated, and the left ICA was then temporarily clamped using a microvascular clip. A small incision was made in the distal part of the left CCA, through which a silicone‐coated monofilament (A5‐283820, Beijing Cinontech Co. Ltd., China) was inserted and advanced along the ICA until mild resistance indicated occlusion of the middle cerebral artery. The left MCA was chosen to ensure consistency with our laboratory's established surgical protocol and to allow consistent assessment of sensorimotor deficits and infarct volume. After 120 min of occlusion, reperfusion was initiated by gently withdrawing the filament. Sham‐operated rats underwent identical surgical procedures except for filament insertion. MCAO model success was confirmed by neurological evaluation at 24 h post‐reperfusion. Animals with a Longa score of 1–3 were included in subsequent analyses. Animals that died during or within 24 h after surgery, displayed no neurological deficits (Longa score = 0), or exhibited signs of subarachnoid hemorrhage upon sacrifice were excluded.

### 2.3 Statistical Analysis

Clinical data analysis: Continuous variables were expressed as mean ± standard deviation (SD) for normally distributed data or median with interquartile range (IQR) for non‐normally distributed data. Normality was assessed using the Shapiro–Wilk test. As the clinical dataset consisted of two groups, normally distributed variables were compared using independent‐samples *t*‐tests, whereas non‐normally distributed variables were analyzed using the Mann–Whitney *U* test. Categorical variables were compared using the chi‐square test or Fisher's exact test, as appropriate. To evaluate the independent association between insomnia and clinical outcomes, multivariable regression models were constructed. Logistic regression was used for binary outcomes and reported as adjusted odds ratios (aORs), while linear regression was applied for continuous outcomes and reported as adjusted mean differences (aMDs). Covariates included age, sex, baseline National Institutes of Health Stroke Scale (NIHSS) score, Alberta Stroke Program Early CT Score (ASPECTS), relevant medical history, and onset‐to‐treatment time.

Experimental data analysis: experimental data are presented as mean ± standard error of the mean (SEM). SD was used for clinical data to describe population‐level variability, whereas SEM was selected for experimental datasets to reflect the precision of mean estimates. Normality was assessed using the Shapiro–Wilk test, and homogeneity of variances was evaluated using Levene's test. For datasets meeting parametric assumptions, one‐way analysis of variance (ANOVA) followed by Tukey's post hoc test was performed. Spearman's rank correlation analysis was used to assess associations between variables.

All statistical analyses were conducted in R, whereas data visualization and figure rendering were completed using GraphPad Prism (version 9.0; GraphPad Software, San Diego, CA, USA). Statistical significance in experimental figures was indicated as *p < 0.05, **p < 0.01, and ***p < 0.001.

Additional methods and materials are described in the Supporting Information.

## 3 Results

### 3.1 Study Population, Demographic Characteristics, Clinical Data and Comparison of Study Endpoints

Baseline characteristics were compared between the two groups. A difference was observed in NIHSS score (17 [13–20.8] vs. 15 [11–19], *p* = 0.026), while the rest of the variables did not show significant differences (*p* > 0.05; Table S1). After adjusting for age, sex, onset‐to‐revascularization time, admission ASPECTS score, intravenous thrombolysis, Trial of Org 10172 in acute stroke treatment (TOAST) classification, and occlusion site, the insomnia group showed worse functional outcomes, with higher 7‐day modified rankin scale (mRS) (5 [3.2–5] vs. 4 [3–5], adjusted MD = 0.2, *p* = 0.007) and 90‐day mRS scores (4 [2–4.8] vs. 3 [1–4], adjusted MD = 0.3, *p* = 0.029; Table S2). The insomnia group also had a higher 90‐day stroke recurrence rate (30.9% vs. 17.0%, adjusted OR = 2.72, *p* = 0.038), while 90‐day mortality and hemorrhagic complications showed no significant differences (*p* > 0.05; Table S2). In laboratory findings, the insomnia group demonstrated increased inflammatory activation, reflected by greater white blood cell count (9.5 [7.9–13.1] vs. 9.3 [7.6–10.9], adjusted MD = 1.7, *p* = 0.022), and a higher neutrophil proportion (85.1% [78.5–90.3] vs. 83.2% [74.4–89.6], adjusted MD = 6.9, *p* = 0.032). D‐dimer levels (1.2 [0.6–2.9] vs. 1.0 [0.5–2.9] mg/L, adjusted MD = 0.09, *p* = 0.474) and fibrinogen levels did not differ significantly between the groups (*p* = 0.980; Table S2).

### 3.2 Sleep Deprivation Worsens Post‐Stroke Outcomes in Antibiotic‐Treated MCAO Rats

**FIGURE 1:**
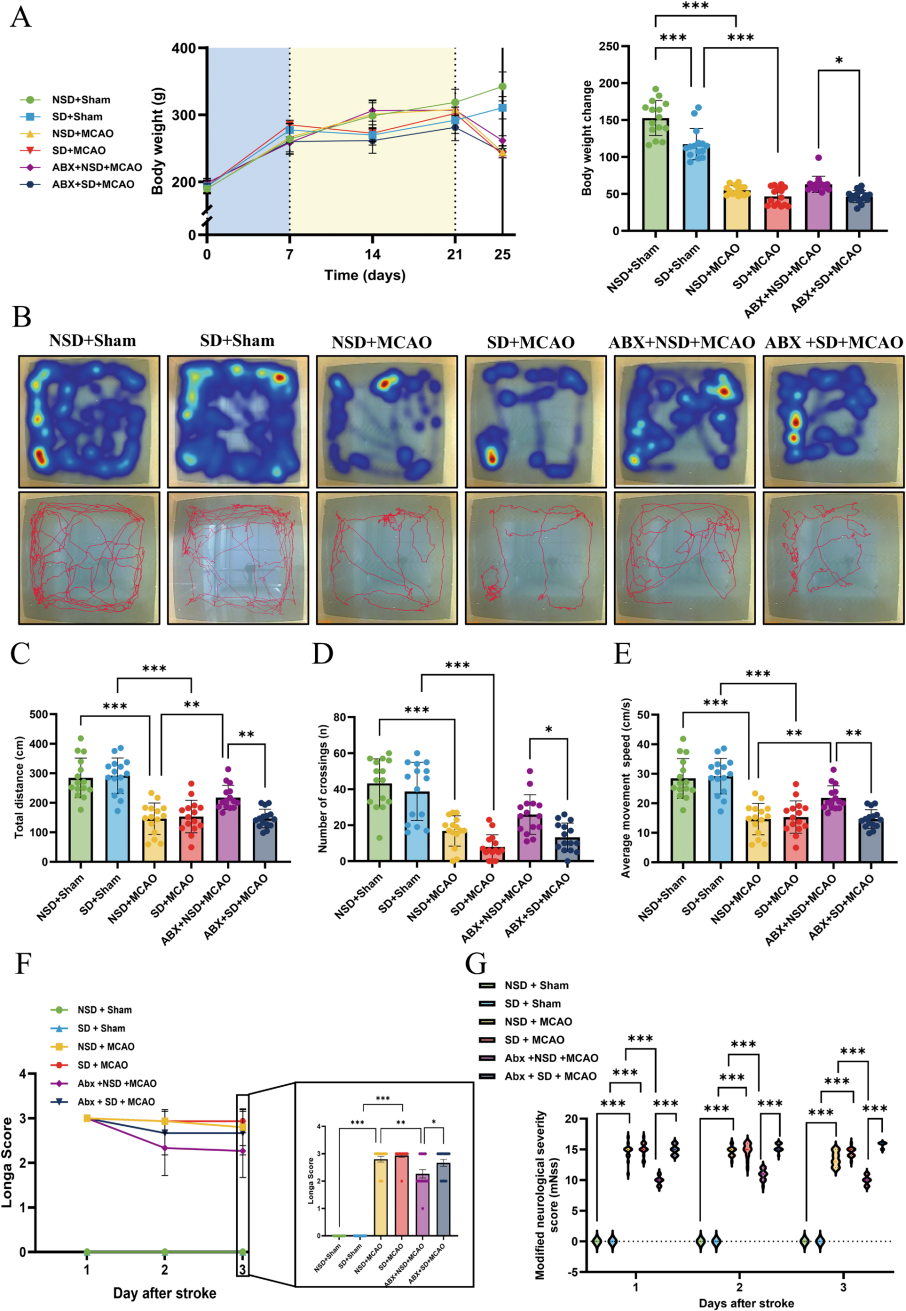
SD aggravates post‐stroke neurological deficits in ABX‐treated rats. (A) In all experimental groups (*n* = 15), the left panel displays the body weight of rats from baseline to 25 days post‐surgery, and the right panel shows the percentage change in body weight on day 25 compared to the baseline. (B) Representative heatmaps and trajectory plots from the OFT illustrate the effects of stroke and SD on spontaneous locomotor activity. (C–E) Quantitative analysis of OFT parameters: (C) total distance moved (*n* = 15), (D) number of grid crossings (*n* = 15), and (E) average speed (*n* = 15). (F) Longa neurological severity scores evaluated at days 1, 2, and 3 post‐stroke (*n* = 15). (G) mNSS assessing the degree of neurological impairment at days 1, 2, and 3 post‐stroke (*n* = 15). Significance levels: *p < 0.05, **p < 0.01, and ***p < 0.001.

The overall experimental design and timeline are illustrated in Figure S1. To investigate the role of SD in shaping post‐stroke outcomes, we compared six experimental groups: NSD + Sham, SD + Sham, NSD + MCAO, SD + MCAO, ABX + NSD + MCAO, and ABX + SD + MCAO. We first observed a significant reduction in body weight in both the SD + Sham and NSD + MCAO groups compared with the NSD + Sham group. Without ABX treatment, body weight did not differ significantly between the NSD + MCAO and SD + MCAO groups. In contrast, we further found that in ABX‐treated MCAO rats, SD significantly alleviated weight loss compared with the NSD condition (Figure 1A). The Open field test (OFT) showed reduced motor and exploratory activity after MCAO, with the NSD + MCAO group displaying marked decreases in motor and exploratory activity, as indicated by locomotor traces confined to the arena periphery (Figure 1B). Quantitative analysis further revealed significant reductions in total distance traveled, number of crossings, and average speed (Figure 1C–E). However, these OFT parameters showed no significant differences between the SD + MCAO and NSD + MCAO groups. Notably, in ABX‐treated rats, the OFT parameters were markedly lower in the ABX + SD + MCAO group than in the ABX + NSD + MCAO group (Figure 1C–E). Consistent with the successful establishment of the model, the NSD + MCAO group exhibited significantly higher longa and modified neurological severity score (mNSS) scores compared with the NSD + Sham group. Similarly, the neurological scores of the SD + MCAO group were significantly higher than those of the SD + Sham group (Figure 1F,G). However, in animals with an intact microbiota, SD under MCAO conditions did not significantly worsen neurological performance compared to the NSD + MCAO group. Notably, in ABX‐treated rats, the ABX + SD + MCAO group displayed significantly higher scores than the ABX + NSD + MCAO group (Figure 1F,G). These findings indicate that SD exacerbates neurological dysfunction after ischemic stroke, but this effect is only evident with ABX treatment.

### 3.3 Sleep Deprivation Aggravates Post‐Stroke Brain Injury in MCAO Rats Under ABX Treatment via Blood–Brain Barrier Disruption

**FIGURE 2:**
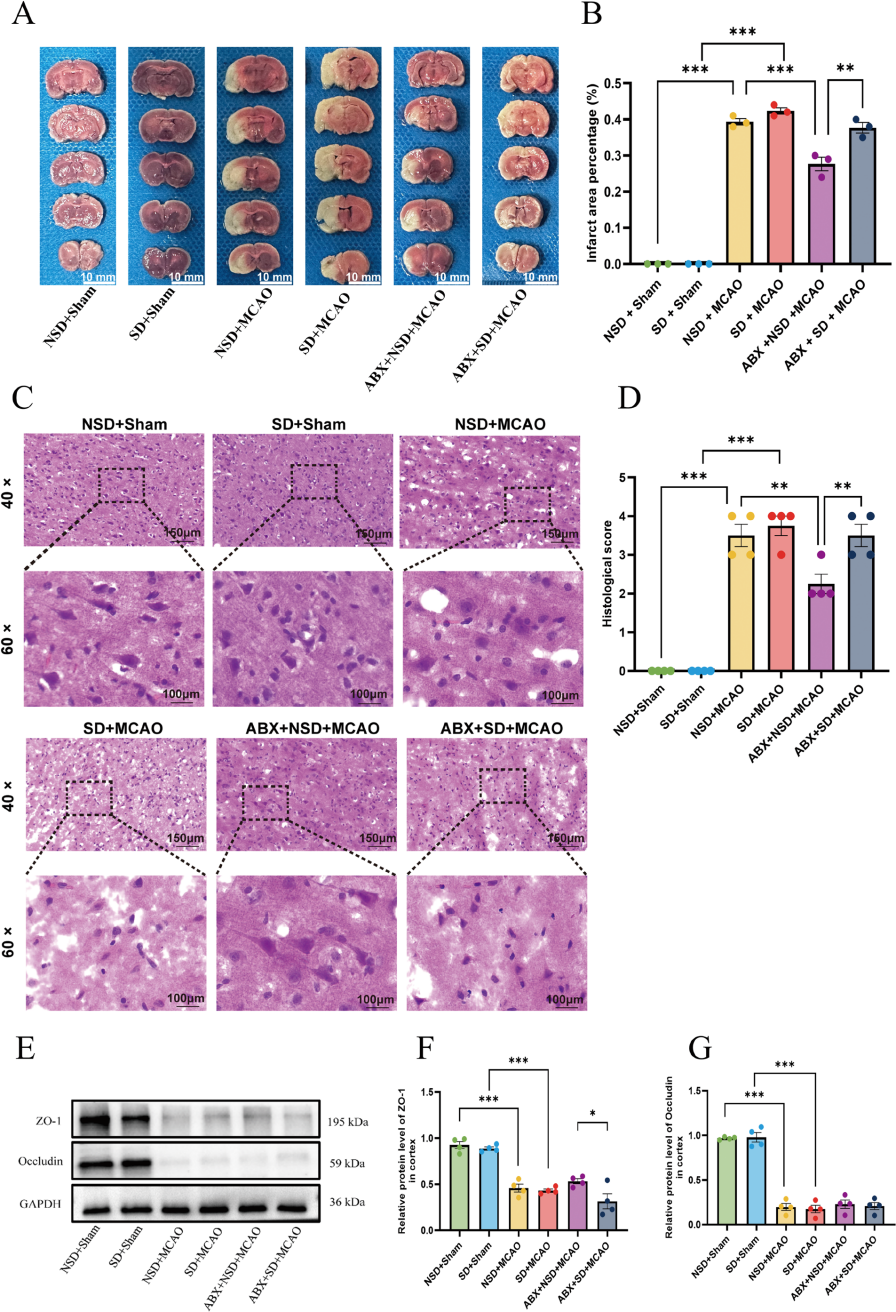
SD exacerbates post‐stroke brain injury and disrupts BBB integrity in ABX‐treated rats. (A) Representative TTC‐stained coronal brain sections showing infarct regions (pale) in the left hemisphere and non‐infarct regions (red) across experimental groups. Images were acquired using a Logitech C920 Pro HD webcam (1920 × 1080 pixels) under standardized lighting conditions. Scale bar = 10 mm. (B) Quantification of infarct volume percentage based on TTC staining (*n* = 3). (C) Representative photomicrographs of the cerebral cortex stained with HE. Scale bar = 150 μm (40×) or 100 μm (60×). (D) Histological score of cortical damage (*n* = 4). (E) Representative images of ZO‐1 and occludin in the cerebral cortex by western blotting. (F) Quantification of relative ZO‐1 protein expression in the cerebral cortex (*n* = 4). (G) Quantification of relative occludin protein expression in the cerebral cortex (*n* = 4). Significance levels: *p < 0.05, **p < 0.01, and ***p < 0.001.

TTC results revealed that, compared with the NSD + Sham group, NSD + MCAO surgery significantly increased infarct volume. Similarly, the SD + MCAO group showed a significant increase in infarct volume compared to the SD + Sham group (Figure 2A,B). Importantly, SD did not exacerbate ischemic brain injury in MCAO rats (NSD + MCAO vs. SD + MCAO). However, in ABX‐treated rats, the ABX + SD + MCAO group exhibited markedly aggravated cerebral damage (Figure 2A,B). These findings were further supported by HE staining and histopathological assessment of the cerebral cortex. The NSD + Sham group showed intact neuronal architecture with well‐preserved cellular morphology, while the SD + Sham group displayed no obvious structural alterations (Figure 2C). In contrast, the NSD + MCAO and SD + MCAO groups showed disorganized cortical arrangement accompanied by extensive neuronal necrosis. Notably, the ABX + SD + MCAO group demonstrated the most severe cortical damage, whereas the ABX + NSD + MCAO group exhibited comparatively attenuated pathological injury (Figure 2C,D). Subsequently, western blot analysis was used to assess BBB tight junction proteins ZO‐1 and occludin. Compared with the NSD + Sham group, the NSD + MCAO group exhibited significant reductions in both ZO‐1 and occludin protein expression. The same was true for the SD + MCAO group compared to the SD + Sham group (Figure 2E–G). Regarding ZO‐1, its expression levels remained unchanged in the SD + MCAO group relative to the NSD + MCAO group under non‐ABX treatment conditions. In contrast, in ABX‐treated rats, ZO‐1 expression was significantly downregulated in the ABX + SD + MCAO group compared with the ABX + NSD + MCAO group (Figure 2F). However, occludin expression showed no significant differences between the ABX + SD + MCAO and ABX + NSD + MCAO groups (Figure 2G).

### 3.4 Sleep Deprivation Compromises Intestinal Barrier Integrity in MCAO Rats Under ABX Treatment

**FIGURE 3:**
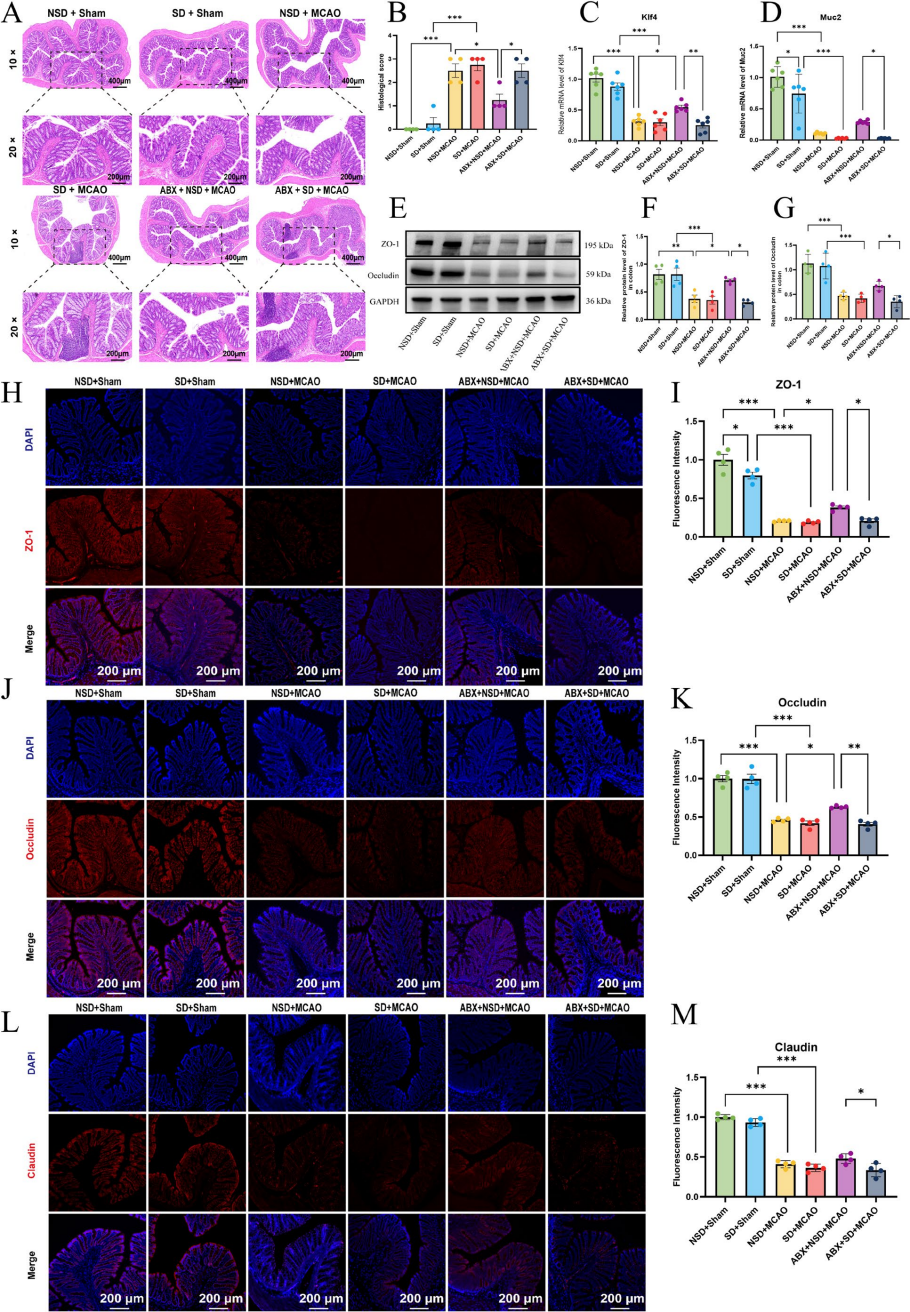
SD exacerbates stroke‐induced intestinal barrier injury in ABX‐treated rats. (A) Representative colon tissue sections stained with HE. (B) Histological score of colonic injury (*n* = 4). (C, D) Relative mRNA expression levels of *Klf4* (C) and *Muc2* (D) in colonic tissue (*n* = 6). (E) Representative images of ZO‐1 and occludin in the colon by western blotting. (F) Quantification of relative ZO‐1 protein expression in the colon (*n* = 4). (G) Quantification of relative occludin protein expression in the colon (*n* = 4). (H, I) Representative immunofluorescence images (H) and quantitative analysis (I) of ZO‐1 expression in colon tissues (*n* = 4). (J, K) Representative immunofluorescence images (J) and quantitative analysis (K) of occludin expression in colon tissues (*n* = 4). (L, M) Representative immunofluorescence images (L) and quantitative analysis (M) of claudin expression (*n* = 4). Significance levels: *p < 0.05, **p < 0.01, and ***p < 0.001.

Given that SD influences stroke outcomes and the gut‐brain axis is pivotal in stroke pathophysiology, we subsequently evaluated morphological and molecular changes in the intestinal barrier. As shown in Figure 3A, compared with the NSD + Sham group, the NSD + MCAO group exhibited severe disruption of the intestinal mucosal architecture, characterized by disorganized crypts, villus atrophy, and inflammatory cell infiltration. Similar intestinal damage was observed in the SD + MCAO group compared to the SD + Sham group. In the absence of ABX treatment, no significant difference in histological scores was observed between the NSD + MCAO and SD + MCAO groups, suggesting that SD alone did not further exacerbate intestinal injury following stroke (Figure 3B). Conversely, rats treated with ABX exhibited severe mucosal damage in the ABX + SD + MCAO group, with histological scores significantly higher than those in the ABX + NSD + MCAO group (Figure 3B). RT‐qPCR supported these findings. Compared with the NSD + Sham group, both *Klf4* and *Muc2* expression were significantly downregulated in the NSD + MCAO group, whereas *Klf4 and Muc2* were markedly reduced in the SD + MCAO group compared with the SD + Sham group. No significant differences in gene expression were observed between the SD + MCAO and NSD + MCAO groups (Figure 3C,D). Figure 3C,D shows that in ABX‐treated rats, *Klf4* and *Muc2* levels were significantly lower in the ABX + SD + MCAO group than in the ABX + NSD + MCAO group. Western blot similarly showed reduced ZO‐1 and occludin expression were significantly reduced in the ABX + SD + MCAO group compared with the ABX + NSD + MCAO group in ABX‐treated rats (Figure 3E–G). No such differential expression was observed in rats without ABX treatment (Figure 3E–G). To further evaluate the spatial distribution and expression of key barrier proteins, IF staining was performed for ZO‐1, occludin, and claudin. In ABX‐treated rats, the ABX + SD + MCAO group showed a marked reduction in all three proteins compared to the ABX + NSD + MCAO group (Figure 3H–M). In contrast, the detrimental effects of SD were not observed in the absence of ABX treatment. Collectively, these findings indicate that SD compromises intestinal barrier integrity specifically in the context of ABX treatment.

### 3.5 Sleep Deprivation Potentiates the Pro‐Inflammatory Effect in MCAO Rats Under ABX Treatment

We measured the levels of multiple inflammatory cytokines in both serum and cortical tissue. The concentrations of IL‐1β, IL‐6, and TNF‐α were significantly higher in the NSD + MCAO group than in the NSD + Sham group in cortical samples. No significant differences were observed between the SD + MCAO and NSD + MCAO groups (Figure S2A–C). Among ABX‐treated rats, all three cytokines were significantly higher in the ABX + SD + MCAO group than in the ABX + NSD + MCAO group (Figure S2A–C). In serum, all three cytokines were significantly higher in the NSD + MCAO group than in the NSD + Sham group. There were no significant differences between the SD + MCAO and NSD + MCAO groups (Figure S2D–F). In ABX‐treated rats, IL‐6 and TNF‐α levels were significantly higher in the ABX + SD + MCAO group than in the ABX + NSD + MCAO group. IL‐1β levels tended to increase in the ABX + SD + MCAO group compared to the ABX + NSD + MCAO group, but this difference was not statistically significant (Figure S2D–F).

### 3.6 Effects of Sleep Deprivation and ABX Treatment on the Structure and Diversity of the Gut Microbiota After Stroke

PCoA based on Bray‐Curtis distances showed no clear separation between the NSD + MCAO and SD + MCAO groups, whereas a distinct cluster pattern was observed between the ABX + NSD + MCAO and the ABX + SD + MCAO groups (Figure S3A). Consistent with this, alpha diversity analysis showed no significant difference between the NSD + MCAO and SD + MCAO groups. In contrast, the Shannon diversity index was significantly lower in the ABX + NSD + MCAO group compared to the NSD + MCAO group (Figure S3B), indicating that ABX treatment reduced microbial diversity. Under SD conditions, both the Shannon and Chao1 indices were significantly higher in the ABX + SD + MCAO group than in the ABX + NSD + MCAO group (Figure S3B,C), suggesting that SD partially restored gut microbial diversity following ABX treatment. At the phylum level, *Firmicutes* and *Bacteroidota* constituted the dominant taxa across all groups, though their relative abundances were substantially influenced by both ABX intervention and SD. In the absence of ABX, the predominance of *Firmicutes* was maintained in the NSD + MCAO group, whereas this microbial balance was disrupted in the SD + MCAO group (Figure S3D). In contrast, compared with the ABX + SD + MCAO group, the ABX + NSD + MCAO group exhibited markedly lower abundances of *Bacteroidota* and *Firmicutes* but higher *Verrucomicrobiota*, indicating profound microbial restructuring. Compared to the NSD + MCAO group, the SD + MCAO group exhibited significant increases in the abundance of *Bacteroidota*, *Proteobacteria*, and *Desulfobacterota*, accompanied by a decrease in *Firmicutes* (Figure S3E,F,H,J). Notably, compared with the ABX + NSD + MCAO group, the ABX + SD + MCAO group showed significantly elevated abundances of *Bacteroidota*, *Firmicutes*, and *Actinobacteriota*, while *Verrucomicrobiota* was considerably reduced (Figure S3E–G,I). A comparison between the ABX + SD + MCAO and SD + MCAO groups revealed that under ABX treatment, SD failed to induce the *Proteobacteria* increase observed in MCAO mice, as shown in Figure S3H.

### 3.7 Sleep Deprivation Reduces *Akkermansia* Abundance and Reshapes Microbial Signatures Under ABX Treatment

**FIGURE 4:**
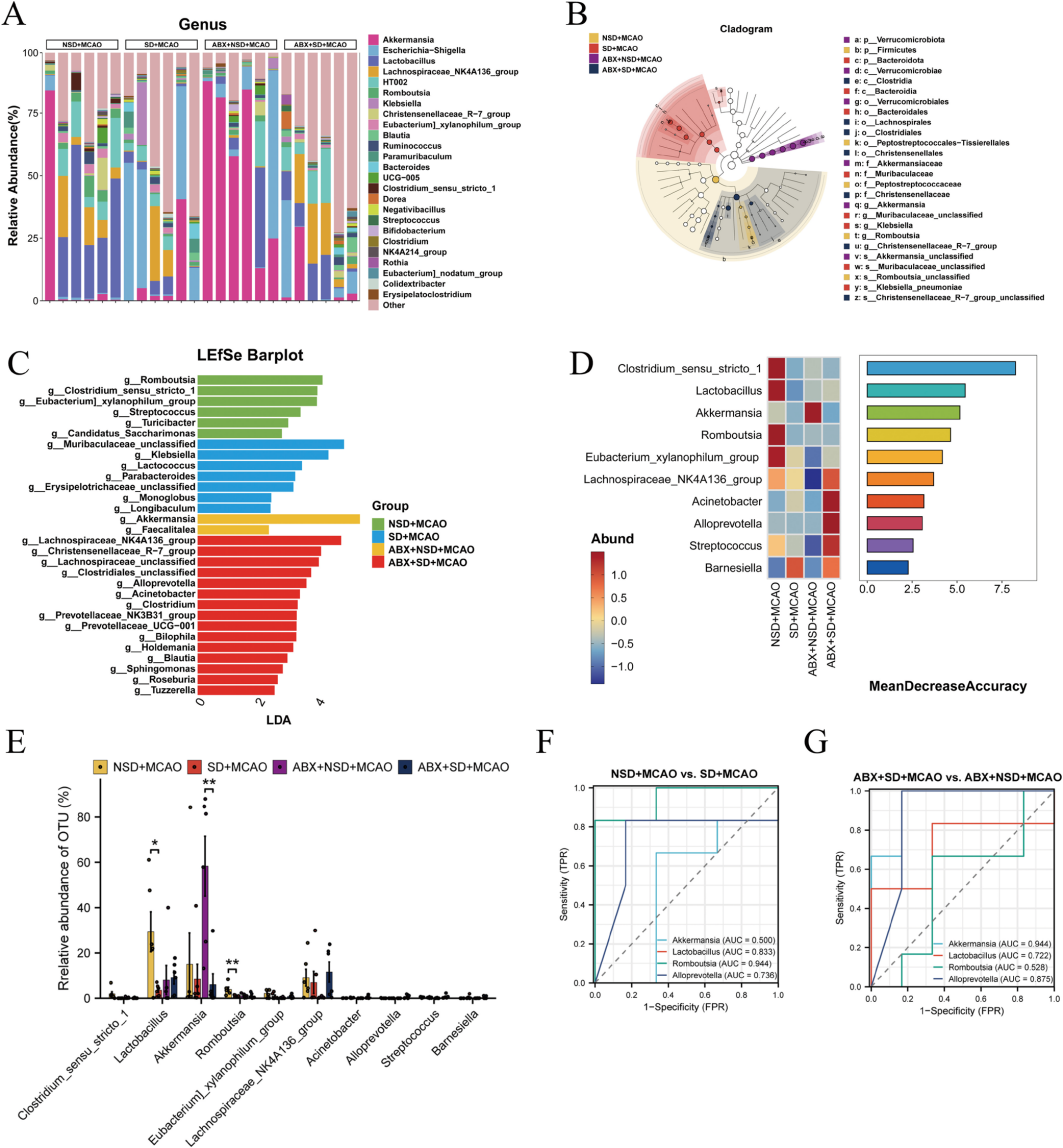
SD modulates gut microbiota composition under ABX and alters the abundance and diagnostic role of *Akkermansia*. (A) Stacked bar chart illustrating genus‐level microbial composition across groups. (B) Cladogram from LEfSe analysis indicating phylogenetic distribution of differentially abundant taxa. (C) LDA score plot displaying taxa with significant abundance differences across groups. (D) Top‐ranked discriminatory genera identified by random forest analysis, along with the corresponding heatmap of their relative abundances. (E) Relative abundance (%) of the 10 most discriminatory genera identified by random forest analysis across four groups (NSD + MCAO, SD + MCAO, ABX + NSD + MCAO, and ABX + SD + MCAO). ROC curves evaluating the discriminatory performance of key genera between NSD + MCAO and SD + MCAO (F), and between ABX + SD + MCAO and ABX + NSD + MCAO (G). Significance levels: *p < 0.05 and **p < 0.01.

To elucidate the impact of SD on gut microbiota composition, we analyzed microbial composition at the genus level. Notable differences were observed among the four groups. Under ABX, *Akkermansia* was enriched in ABX + NSD + MCAO, whereas SD (ABX + SD + MCAO) diminished this enrichment (Figure 4A). The cladogram revealed distinct group‐specific microbial signatures, including enrichment of *Peptostreptococcaceae* and *Romboutsia* in NSD + MCAO, enrichment of *Muribaculaceae* and *Klebsiella* in SD + MCAO, enrichment of *Akkermansia* and other members of the *Akkermansiaceae* family in ABX + NSD + MCAO, and increased abundance of the *Christensenellaceae_R−7_group* and *Clostridia* in ABX + SD + MCAO (Figure 4B). The linear discriminant analysis (LDA) scores further indicated that several genera from the Firmicutes phylum, including the *Clostridium_sensu_stricto_1* and *Romboutsia*, were dominant in the NSD + MCAO group, while *Lactococcus* and *Muribaculaceae* were enriched in the SD + MCAO group, *Akkermansia* and *Faecalitalea* were enriched in ABX + NSD + MCAO, and *Lachnospiraceae_NK4A136‐_group* and *Clostridiales* were enriched in the ABX + SD + MCAO group (Figure 4C). Using a random forest algorithm, we identified ten key discriminatory genera that served as major biomarkers for distinguishing the four groups. Among these, *Clostridium_sensu_stricto_l*, *Lactobacillus*, *Akkermansia*, and *Romboutsia* were ranked highest in importance based on the mean decrease accuracy metric (Figure 4D). As shown in Figure 4E, the bar chart illustrates the relative abundance changes of the 10 key differential genera identified by random forest analysis across different treatment groups. Compared with the SD + MCAO group, the NSD + MCAO group showed a higher abundance of *Lactobacillus* and *Romboutsia*. Under ABX treatment, the ABX + NSD + MCAO group showed higher *Akkermansia* abundance than the ABX + SD + MCAO group. Conversely, SD in the ABX + SD + MCAO group resulted in increased *Alloprevotella* abundance compared to the ABX + NSD + MCAO group (Figure 4E). ROC curve analysis was used to evaluate the discriminatory performance of these key genera. In the comparison between NSD + MCAO and SD + MCAO (Figure 4F), *Romboutsia* exhibited the highest discriminatory power (AUC = 0.944), followed by *Lactobacillus* (AUC = 0.833) and *Alloprevotella* (AUC = 0.736). However, *Akkermansia* showed no significant discriminatory ability (AUC = 0.500). In contrast, in the comparison between the ABX + SD + MCAO and ABX + NSD + MCAO groups, *Akkermansia* (AUC = 0.944) and *Alloprevotella* (AUC = 0.875) demonstrated strong discriminatory power, whereas *Lactobacillus* (AUC = 0.722) showed only moderate performance, and *Romboutsia* (AUC = 0.528) exhibited negligible discriminatory capacity (Figure 4G). Together, these results show that ABX reshapes SD‐related microbial features, with *Akkermansia* becoming the key discriminative genus under ABX treatment.

### 3.8 Sleep Deprivation Drives Metabolic Reprogramming in MCAO Rats Under ABX Treatment

**FIGURE 5:**
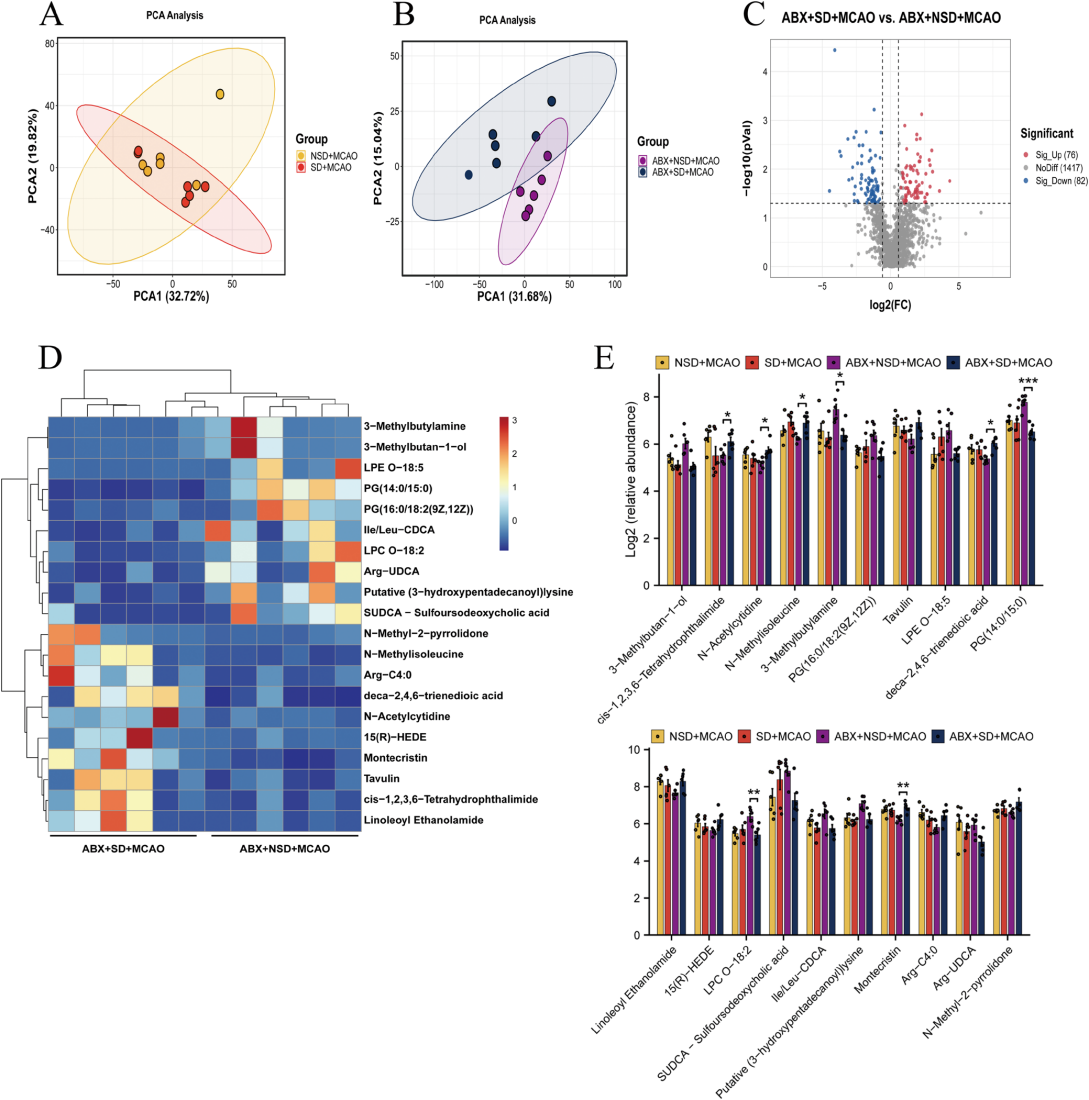
Metabolomic analysis reveals key metabolites altered by SD under ABX treatment. (A, B) PCA score plots derived from LC–MS data show overall metabolic differences among groups. (C) Volcano plot showing differential metabolites between the ABX + SD + MCAO and ABX + NSD + MCAO groups. Red dots represent significantly upregulated (*n* = 76); blue dots indicate significantly downregulated (*n* = 82), and gray dots denote metabolites with no significant change (*n* = 1417). (D) Heatmap of the top 20 most altered metabolites ranked by |Log_2_FC| between ABX + SD + MCAO and ABX + NSD + MCAO. Colors reflect relative abundance (red, high; blue, low). (E) Quantitative comparison of the relative abundances of the top 20 differential metabolites among the four groups (NSD + MCAO, SD + MCAO, ABX + NSD + MCAO, ABX + SD + MCAO). Significance levels: *p < 0.05, **p < 0.01, and ***p < 0.001.

Principal component analysis (PCA) assessed metabolic differences among the four groups. The results showed that the metabolic profiles of NSD + MCAO and SD + MCAO highly overlapped (Figure 5A). In contrast, a clear separation was observed between groups subjected to ABX treatment (ABX + NSD + MCAO vs. ABX + SD + MCAO), indicating that the metabolic impact of SD was more pronounced without an intact gut microbiota (Figure 5B). Differential metabolite analysis identified SD‐associated metabolic shifts under ABX, revealing158 metabolites were significantly altered, including 76 upregulated and 82 downregulated in the ABX + SD + MCAO group (Figure 5C). A clustered heatmap displayed the top 20 metabolites with the largest |Log_2_FC| between ABX + SD + MCAO and ABX + NSD + MCAO. Among these, N‐Methyl‐2‐pyrrolidone, N‐Methylisoleucine, and cis‐1,2,3,6‐Tetrahydrophthalimide were substantially elevated in the ABX + SD + MCAO group (Figure 5D,E), suggesting that SD under ABX treatment enhances metabolic pathways related to methylated compounds, branched‐chain amino acid derivatives, and phthalimide metabolites. Quantitative comparison of the top 20 metabolites confirmed the patterns observed in the heatmap. N‐Acetylcytidine, N‐Methylisoleucine, cis‐1,2,3,6‐Tetrahydrophthalimide, Tavulin, deca‐2,4,6‐trienedioic acid, and Montecristin were significantly elevated in the ABX + SD + MCAO group compared to the ABX + NSD + MCAO group (Figure 5D), supporting the enhancement of methylated compound metabolism, branched‐chain amino acid derivative metabolism, phthalimide‐related pathways, and possibly alkaloid‐associated metabolic processes. Conversely, 3‐Methylbutylamine, PG(14:0/15:0), and LPC O‐18:2 were significantly decreased (Figure 5E), indicating suppression of specific amine and lysophosphatidylcholine‐mediated lipid metabolic pathways under SD in the context of ABX treatment.

### 3.9 Associations of *Akkermansia* Abundance With Key Metabolites and Stroke Severity

**FIGURE 6:**
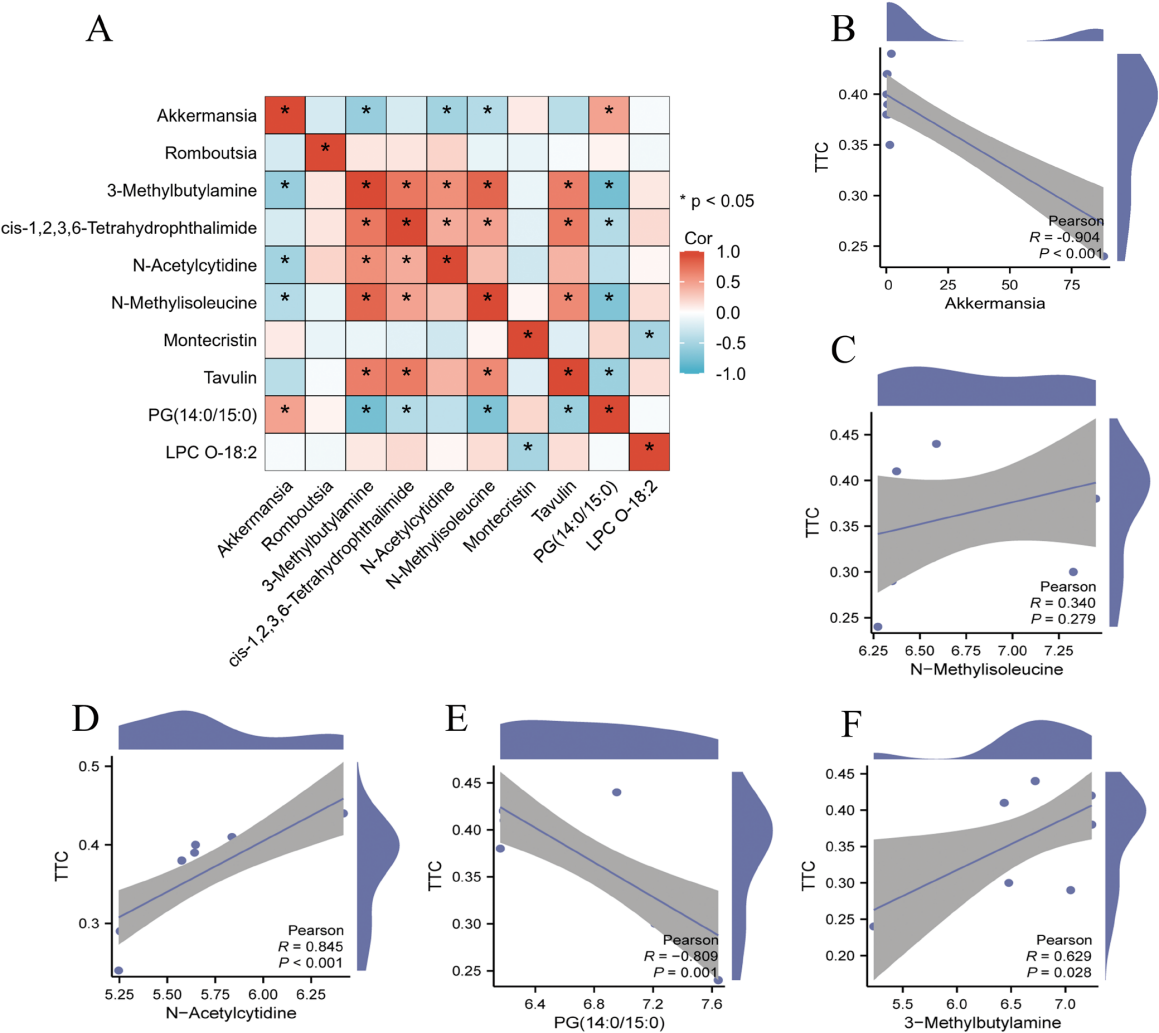
Spearman correlation analysis among key microbial genera, differential metabolites, and infarct volume (measured by TTC). (A) Heatmap of Spearman correlations between microbial genera and representative differential metabolites. (B–F) Scatter plots showing correlations of infarct volume with *Akkermansia* abundance (B), N‐Methylisoleucine (C), N‐Acetylcytidine (D), PG(14:0/15:0) (E), and 3‐Methylbutylamine (F). Correlation strength is indicated by Spearman's *r* value with 95% confidence intervals. Significance levels: *p < 0.05.

To clarify the role of *Akkermansia*, we examined correlations between its abundance, key metabolites, and indicators of stroke severity. A correlation heatmap revealed a strong negative correlation between *Akkermansia* abundance and several stroke‐related metabolites (Figure 6A). In particular, *Akkermansia* abundance correlated negatively with 3‐Methylbutylamine, N‐Acetylcytidine, and N‐Methylisoleucine, and correlated positively with PG(14:0/15:0). Subsequently, Spearman analysis was used to explore relationships among *Akkermansia* abundance, key metabolites, and stroke severity measured by infarct volume via TTC. As shown in Figure 6B–F, *Akkermansia* abundance showed a strong negative correlation with infarct volume (*r* = −0.904, *p* < 0.001), indicating a potential protective role against stroke injury (Figure 6B). In contrast, N‐Methylisoleucine showed no significant correlation with infarct volume (*r* = 0.340, *p* = 0.279; Figure 6C). N‐Acetylcytidine was positively correlated with infarct volume (*r* = 0.845, *p* < 0.001), suggesting a possible exacerbating effect on stroke injury (Figure 6D). Similarly, PG(14:0/15:0) exhibited a negative correlation with infarct volume (*r* = −0.809, *p* = 0.001), implying a protective function in stroke recovery (Figure 6E). Lastly, 3‐Methylbutylamine displayed a weak positive correlation with infarct volume (*r* = 0.629, *p* = 0.028), suggesting a potential contribution to stroke severity (Figure 6F).

## 4 Discussion

Our findings indicate that sleep disturbance is associated with unfavorable stroke outcomes and that its detrimental impact on brain injury depends on the state of the gut microbiota. By integrating clinical observations with behavioral, histological, molecular, microbiome, and metabolomic analyses, we found that post‐stroke indices were largely comparable when microbial homeostasis was preserved. However, under ABX‐induced dysbiosis, SD amplified neuroinflammation relative to controls, disrupted intestinal and BBB integrity, and worsened functional deficits. 16S rRNA sequencing and untargeted metabolomics further revealed a reduction in *Akkermansia*, accompanied by a shift toward specific proinflammatory taxa and altered metabolic profiles. Correlation analyses showed that lower *Akkermansia* abundance was associated with larger infarct volumes and poorer neurological recovery. Collectively, these results suggest that maintaining microbial stability—particularly sustaining *Akkermansia* colonization and function—may buffer against sleep‐disruption–related inflammatory escalation and represent a feasible strategy to improve post‐stroke prognosis.

Sleep plays a crucial role in maintaining neural homeostasis [30, 31, 32]. Accumulating evidence indicates that sleep disturbances are not merely a concomitant symptom of stroke but an independent risk factor influencing the trajectory of post‐stroke recovery [33, 34]. Consistent with this notion, our clinical observations showed that AIS patients with insomnia exhibited slower neurological recovery after mechanical thrombectomy and higher stroke recurrence rates during follow‐up. These patients also exhibited heightened inflammatory and prothrombotic responses, such as elevated leukocyte counts. Although insomnia frequently coexists with conditions like hypertension and diabetes—both more prevalent in our insomnia cohort—the association between insomnia and these metabolic/circulatory risk factors did not reach statistical significance, leaving its specific contribution to post‐stroke pathology an open question.

The homeostasis of the gut microbiota and its metabolites has attracted increasing attention as a key regulator of peripheral immunity and central nervous system injury responses [18, 35, 36]. Evidence indicates that the gut microbiota dysbiosis alters peripheral immune activation and the production of inflammatory mediators [37, 38, 39]. It also disrupts the structural integrity of the intestinal mucosal barrier, thereby promoting the entry of bacteria‐derived endotoxins and pro‐inflammatory factors into the circulation and compromising BBB function [40, 41, 42].

To further clarify whether gut microbiota mediates the effect of sleep disturbances on stroke, we evaluated the impact of SD on ischemic injury under different microbiota conditions. When the gut microbiota was intact, SD exerted minimal impact on post‐stroke outcomes, as indicated by comparable body weight, locomotor activity, neurological scores (Longa and mNSS), infarct volume, and expression of BBB tight junction proteins ZO‐1 and occludin. However, when microbial homeostasis was disrupted, SD markedly exacerbated neurological and motor deficits, and caused more severe damage to both brain and intestinal tissues. This is consistent with previous reports that gut dysbiosis significantly worsens neurological outcomes and amplifies inflammatory responses following brain injury, largely due to disruption of intestinal barrier integrity [18, 19]. Under dysbiotic conditions, SD intensified neuroinflammation, as characterized by larger infarcts, elevated inflammatory cytokines, and reduced tight junction protein levels. Collectively, these results reveal that the detrimental impact of SD on ischemic injury is modulated by the gut microbiota; it is minimal when microbial homeostasis is preserved but significantly amplified upon its disruption.

We next performed 16S rRNA sequencing to define the microbial basis underlying the differential regulation of intestinal epithelial barrier integrity. When the microbiota were impaired, SD‐induced alterations were characterized by a marked reduction of *Akkermansia*, accompanied by a relative enrichment of pro‐inflammatory taxa such as *Lachnospiraceae* and *Clostridia*. *Akkermansia*, a mucus‐associated symbiont, is known to maintain mucosal barrier structure, support tight junction protein expression, and regulate epithelial immune homeostasis. Thus, reduced abundance reflects weakened barrier defense and increased inflammatory susceptibility [43, 44, 45]. Previous studies have shown that SD reduces *Akkermansia* levels, whereas melatonin supplementation can restore its abundance and improve intestinal epithelial barrier function and neuroinflammatory status [46, 47]. Moreover, increased *Akkermansia* abundance has been associated with reduced BBB permeability and decreased microglial activation in models of Alzheimer's disease and Parkinson's disease [48, 49], suggesting a broader neuromodulatory role. Taken together, the observed transition from a barrier‐supportive microbiota configuration to an inflammation‐prone state suggests that *Akkermansia* may represent a critical microbial node linking sleep states to stroke pathology. Although our study identified *Akkermansia* depletion as a correlate of SD‐exacerbated stroke injury, direct causal validation within our model remains incomplete. However, accumulating evidence from supplementation, metabolite intervention, fecal microbiota transplantation, and Mendelian randomization studies strongly supports a protective role of *Akkermansia* in ischemic stroke [50, 51, 52, 53, 54]. These findings suggest that *Akkermansia* depletion in our SD model is likely a functionally relevant contributor to worsened ischemic injury rather than a purely descriptive association.

Metabolomic profiling was conducted to evaluate the functional consequences of microbial restructuring. Under microbiota‐impaired conditions, SD triggered a distinct metabolic reprogramming, characterized by elevated N‐Acetylcytidine and N‐Methylisoleucine—markers of enhanced RNA modification and stress‐associated amino acid metabolism. In ischemic stroke, such elevations in methylation‐related and branched‐chain amino acid associated metabolites have been linked to pro‐inflammatory microglial activation and astrocytic reactivity, signifying an immunometabolic shift toward a glycolytic, inflammatory state [55, 56, 57]. In contrast, levels of 3‐Methylbutylamine, PG14:0/15:0, and LPC O‐18:2 were markedly reduced, signaling a breakdown in microbiota‐host communication (3‐Methylbutylamine), diminished microbial membrane lipid support (PG14:0/15:0), and compromised membrane repair and neurotransmitter precursor availability (LPC O‐18:2). These impairments in lipid and amine metabolism, linked to poor cellular resilience and neural recovery [58, 59], correspond with the larger infarct size, heightened inflammation, and worse behavioral outcomes. Notably, *Akkermansia* abundance negatively correlated with infarct volume and neurological deficits, and positively correlated with membrane‐supportive lipids, suggesting that it is a crucial node in the metabolic network that buffers against SD‐induced injury exacerbation. Our metabolomic findings resonate with emerging evidence highlighting the critical role of acetate in mediating the interplay between sleep, metabolism, and brain injury. A recent study demonstrated that acetate accumulation in hypothalamic astrocytes represents a defensive response against sleep disruption, protecting metabolic fitness and cognitive performance by restoring glycolytic and tricarboxylic acid cycle activity [17]. Complementarily, acetate has been shown to exert direct neuroprotective effects in ischemic stroke by stimulating the astrocyte‐neuron lactate shuttle and promoting tau protein lactylation, thereby enhancing neuronal plasticity and reducing infarct volume [60]. Notably, *Akkermansia muciniphila* is a major short‐chain fatty acid producer, and its depletion in our ABX + SD + MCAO model may have led to reduced acetate availability, thereby compromising both the metabolic buffering capacity against SD‐induced stress and the neuroprotective lactate shuttle. This interpretation provides a mechanistic link between our observation of *Akkermansia* depletion, metabolic dysregulation, and worsened stroke outcomes, and suggests that acetate‐mediated pathways may represent actionable therapeutic targets. Our findings should be interpreted within the broader framework of central‐peripheral cross‐talk in brain diseases. As recently reviewed, blood‐derived factors serve as critical mediators of inter‐organ communication, transmitting metabolic, immune, and inflammatory signals between the gut, liver, and brain [15]. In the context of our study, SD‐induced gut dysbiosis, particularly *Akkermansia* depletion, likely altered the composition of blood‐borne metabolites and immune mediators, which in turn exacerbated BBB disruption, neuroinflammation, and infarct expansion. The elevated D‐dimer levels, leukocyte counts, and neutrophil proportions observed in our clinical insomnia cohort further support the notion that peripheral inflammatory and prothrombotic signals contribute to central nervous system injury. Thus, both our clinical and experimental data converge to demonstrate that SD worsens stroke outcomes through a pathway involving peripheral to central signaling, adding to the growing body of evidence supporting the importance of central‐peripheral interactions in determining stroke prognosis.

In summary, this study provides clinical and experimental evidence that sleep state is a critical determinant of post‐stroke outcomes. Clinically, insomnia is associated with poorer long‐term neurological recovery, alongside heightened inflammatory responses. Experimentally, SD significantly aggravates brain injury, BBB disruption, and neuroinflammation in the context of gut microbiota dysbiosis. These pathological changes are linked to a reduction in *Akkermansia* abundance and the activation of pro‐inflammatory metabolic pathways. Our findings underscore the gut microbiota's pivotal role in mediating the impact of sleep disruption on ischemic stroke. Specifically, a stable ecosystem provides the host with resilience against sleep‐loss‐induced inflammatory amplification, whereas the loss of this homeostasis ablates such protective buffering. Consequently, therapeutic strategies aimed at maintaining or restoring gut microbial equilibrium—potentially through targeting *Akkermansia*—hold promise for improving stroke outcomes in sleep‐disturbed individuals.

This study has several limitations. First, the generalizability of the clinical findings is constrained by the retrospective design and a limited sample size, notwithstanding the multicenter origin of the data. Future prospective studies with larger cohorts are required. This study was conducted only in male rats to minimize hormonal variability. Nevertheless, sex differences in ischemic injury and neuroinflammation should be explored in future studies including both sexes. Third, although converging evidence from independent studies—including *Akkermansia* gavage supplementation [51, 53], *Akkermansia*‐derived metabolite intervention [52], fecal microbiota transplantation [50], and Mendelian randomization [54]—strongly supports a causal role for *Akkermansia* in ischemic stroke outcomes, direct interventional validation within our specific SD‐MCAO paradigm was not performed due to current laboratory constraints. Future studies incorporating *Akkermansia* supplementation, mono‐colonization, or targeted metabolite inhibition are warranted to definitively confirm causality in the context of sleep deprivation‐associated stroke exacerbation. Finally, the assessment was limited to short‐term outcomes. A longer follow‐up period is essential to fully understand the long‐term impact of SD and gut microbiota composition on neurological recovery.

## Author Contributions

X.N. led the conceptualization and data analysis; S.‐Y.Z., L.Y., L.‐L.T., X.‐Y.M., and Y.‐M.X. performed experimental work; C.‐M.Q. and W.‐J.Z. analyzed data; C.‐M.Q., Y.‐Q.S., and C.‐S.L. contributed to study design, and C.‐S.L. approved the final manuscript.

## Funding

This study was supported by Wuxi Municipal Health Commission (Grant MS201955).

## Disclosure

The authors have nothing to report.

## Ethics Statement

All clinical procedures were reviewed and approved by the Ethics Committee of the Affiliated Hospital of Jiangnan University (Approval No. MR‐32‐24‐055045). The study was conducted in accordance with the Declaration of Helsinki (revised in 2013). All animal experiments were reviewed and approved by the Animal Experimentation Ethics Committee of Jiangnan University (Approval No. JN. 20240315S0900515) and were performed in accordance with the National Institutes of Health Guide for the Care and Use of Laboratory Animals.

## Consent

The requirement for informed consent was waived, as the study employed a retrospective design and utilized de‐identified medical record data without any direct patient involvement. The research protocol received approval from the institutional ethics committee, and strict confidentiality procedures were applied throughout data collection and analysis.

## Conflicts of Interest

The authors declare no conflicts of interest.

## Supporting information


**Data S1:** Supplementary Materials and Methods

**Figure S1:** Overview of animal treatments and experimental design.

**Figure S2:** SD potentiates central and peripheral pro‐inflammatory responses in ABX‐treated rats. (A–C) Quantitative analysis of IL‐1β (A), IL‐6 (B), and TNF‐α (C) protein levels in the cerebral cortex by ELISA (*n* = 6). (D–F) Quantitative analysis of IL‐1β (D), IL‐6 (E), and TNF‐α (F) protein levels in serum by ELISA (*n* = 6). Significance levels: *p < 0.05, **p < 0.01, and ***p < 0.001.

**Figure S3:** ABX treatment significantly alters gut microbial diversity and composition after stroke, but its regulatory effect is attenuated under SD. (A) PCoA based on Bray‐Curtis distances. (B, C) Alpha diversity indices of the gut microbiota, including the Chao1 and Shannon indices. (D) Relative abundances of microbial taxa at the phylum level across experimental groups. (E–J) Comparative analyses of the relative abundances of major bacterial phyla: Bacteroidota, Firmicutes, Verrucomicrobiota, Proteobacteria, Actinobacteriota, and Desulfobacterota among different experimental groups. Significance levels: *p < 0.05, **p < 0.01, and ***p < 0.001.

**Table S1:** Baseline characteristics of the patients.

**Table S2:** Comparison of study endpoints.

## Data Availability

The data used to support the findings of this study are available from the corresponding author upon request.
