# Supplementary material for: Sleep Deprivation Exacerbates Ischemic Stroke Outcomes via Akkermansia Depletion and Metabolic Dysregulation
Source: CNS Neurosci Ther. 2026 May 20;32(5):e70933. doi: 10.1002/cns.70933 (PMC13240125; doi:10.1002/cns.70933)
Supplement: Supplementary file 1 — Data S1: Supplementary Materials and Methods [file CNS-32-e70933-s002.docx]

**Supporting Information**

**Sleep Deprivation Exacerbates Ischemic Stroke Outcomes via Akkermansia Depletion and Metabolic Dysregulation**

Xin Nie^1, 2^, Sheng-Yang Zhou^2, 3^, Le Yao^1^, Lu-Lu Tan^2, 3^, Xiao-Yu Ma^2, 3^, Yi-Meng Xia^2, 3^, Chun Cui^2, 3^, Wei-Jiang Zhao^2, 3^, Chen-Meng Qiao^2, 3^, Yan-Qin Shen^2, 3^, Chao-Sheng Li^1, 2*^

^1^Department of Neurology, Affiliated Hospital of Jiangnan University, Wuxi, Jiangsu 214122, China.

^2^Lab of Neurodegeneration and Injury, Wuxi School of Medicine, Jiangnan University, No. 1800, Lihu Avenue, Binhu District, Wuxi 214122, China.

^3^MOE Medical Basic Research Innovation Center for Gut Microbiota and Chronic Diseases, School of medicine, Jiangnan university, Wuxi, Jiangsu 214122, China.

*Correspondence to: Chao-Sheng Li.

E-mail address: [Wxsylcs@163.com](mailto:Wxsylcs@163.com)

**Materials and Methods**

**Open Field Test (OFT)**

As described by Prut and Belzung[1], the OFT is a classic behavioral method used to assess a rodent’s spontaneous locomotor activity, exploratory behavior, and anxiety-like behavior. Each rat was placed individually into an opaque open-field arena (50 cm × 50 cm × 50 cm), the floor of which was divided into nine equal grids (16.7 cm × 16.7 cm). Each session was video-recorded for 5 min. The recordings were subsequently analyzed with EthoVision software (Noldus, Wageningen, Netherlands) to quantify total distance traveled, average velocity, and number of grid crossings.

**Neurological Scoring and Infarct Volume Assessment**

Neurological deficits in rats with AIS were evaluated using two established scoring systems: the Longa neurological score and the modified Neurological Severity Score (mNSS). The Longa score provides a global assessment of neurological function, whereas the mNSS specifically examines motor, sensory, balance, and reflex functions. Evaluations were performed on days 1, 2, and 3 following MCAO surgery. Detailed scoring criteria were applied in accordance with previously published protocols[2].

Infarct volume was measured using 2,3,5-triphenyltetrazolium chloride (TTC, T8877, Sigma-Aldrich, USA) staining. Briefly, brains were sectioned coronally into 2-mm-thick slices. The sections were incubated in a 2% TTC solution at 37 °C in the dark for 20 min and subsequently fixed in 4% paraformaldehyde (PFA). Non-infarct areas stained red while infarct areas stained off-white. Following staining, the brain slices were placed on a blue background for imaging and scanning. The infarct areas were quantified using ImageJ software. To correct for cerebral edema, infarct volume was calculated using the following formula: Infarct volume (%) = [(contralateral hemisphere volume – non-infarct ipsilateral hemisphere volume)/contralateral hemisphere volume] × 100%.

**Hematoxylin and Eosin (HE) Staining**

Rats were deeply anesthetized with 2–3% isoflurane (S190815, Yuyan, China) and then transcardially perfused with ice-cold phosphate-buffered saline (PBS), followed by 4% PFA. After perfusion, the brains and colon segments were immediately dissected and post-fixed in 4% PFA for an additional 24 h at 4℃. The fixed tissues were subsequently processed through a standard paraffin-embedding protocol, which included dehydration in a graded ethanol series and clearing in xylene. Paraffin-embedded tissue blocks were sectioned serially at a thickness of 5 μm. For HE staining, sections were deparaffinized, rehydrated, and stained with hematoxylin for 5 min, immersed in alcohol for 1 min, followed by eosin for 15 s. Subsequently, the sections were re-immersed in alcohol and xylene. Tissue morphology and structure were then examined under a light microscope, and representative images were captured.

**Immunofluorescence (IF) Staining**

Paraffin-embedded blocks were cut into 5 μm-thick serial sections. The sections were floated onto adhesive glass slides in a 45℃ water bath and subsequently baked at 60℃ for 2 h. For staining, the slides were deparaffinized in xylene and rehydrated through a graded ethanol series (100% to 75%). Heat-induced antigen retrieval was performed by incubating the sections in citrate buffer (pH 6.0) at 95℃ for 20 min. After cooling to room temperature, non-specific binding was blocked with 10% normal sheep serum for 30 min. The sections were then incubated overnight at 4℃ with the following primary antibodies: rabbit anti-ZO-1 (1:200, 21773-1-AP, Proteintech, USA), rabbit anti-occludin (1:200, 27260-1-AP, Proteintech, USA), and rabbit anti-claudin (1:200, 13050-1-AP, Proteintech, USA). The sections were washed three times in PBS and incubated with a Cy3-conjugated goat anti-rabbit IgG secondary antibody (1:500, A0516, Beyotime, China) at 37°C for 1 h. Cell nuclei were stained with DAPI. Finally, the sections were imaged under a fluorescence microscope (Carl Zeiss LSM880, Zeiss, Germany).

**Western Blotting**

Total proteins were extracted from the cerebral cortex and colon tissue. Tissue samples (10 mg) were homogenized in 100 μL of RIPA lysis buffer (Beyotime, China) containing 1% phenylmethanesulfonyl fluoride (PMSF) (Beyotime, China). The tissue was then homogenized using a tissue homogenizer, followed by centrifugation at 4°C for 10 min. The supernatant was collected for further analysis. A BCA protein assay kit (Biosharp, China) was used to determine the protein concentration. The protein samples were heated at 100°C for 5 min. The samples (25 μg) were separated using 10% or 12.5% sodium dodecyl sulfate-polyacrylamide gel electrophoresis and transferred to a PVDF membrane (Millipore, USA). The membrane was then blocked with 5% non-fat milk and incubated with primary antibodies at 4°C overnight. The following primary antibodies were used: rabbit anti-ZO-1 (1:5000, 21,773-1-AP, Proteintech, USA), rabbit anti-occludin (1:1000, 27,260-1-AP, Proteintech, USA), and rabbit anti-GAPDH (1:1000, 10,495-1-AP, Proteintech, USA). After washing, the membrane was incubated with a horseradish peroxidase-conjugated goat anti-rabbit IgG secondary antibody (1:10000, BA1054, Boster, China) at room temperature for 2 h. Signals were then visualized using an ECL detection reagent (36208ES76, Yeasen, China). The output images were analyzed using ImageJ (NIH, Bethesda, USA).

**Quantitative Real-Time PCR**

Total RNA was extracted from brain and colon tissues with a commercial RNA isolation kit following the manufacturer’s instructions. cDNA was synthesized from total RNA using the HiScript III All-in-one RT SuperMix kit. Quantitative real-time PCR was performed using a SYBR Green qPCR master mix with the LightCycler 480 system. GAPDH served as the internal reference gene. Relative gene expression levels were calculated using the 2^−ΔΔCT^ method. The primer sequences used were as follows: Rat *Klf4*, forward: 5′-TATACATTCCGCCACAGCAG-3′, reverse: 5′-TGGGCTTC-CTTTGCTAACAC-3′; rat *Muc2*, forward: 5′-TCCCTCTTACAAGGGCAATG-3′, reverse: 5′-TTCCAGCTGTTCCCA-AAGTC-3′.

**Enzyme-Linked Immunosorbent Assay (ELISA)**

At the experimental endpoint, rats were euthanized, and whole blood was collected via cardiac puncture. The cerebral cortex was rapidly dissected on ice. Blood samples were left to clot at room temperature for 30 min, followed by centrifugation at 3000 rpm for 10 min at 4°C to isolate serum. Simultaneously, the dissected cerebral cortex was homogenized on ice in RIPA lysis buffer supplemented with a protease inhibitor cocktail. The homogenate was then centrifuged, and the supernatant was collected. Total protein concentration was determined using a BCA Protein Assay Kit (Biosharp, China). The concentrations of IL-1β (EK0393, Boster, China), IL-6 (EK0412, Boster, China), and TNF-α (EK0526, Boster, China) in both serum and cortical homogenates were quantified using commercial ELISA kits according to the manufacturer’s instructions.

**16S rRNA Gene Sequencing**

To investigate the gut microbiota, fresh fecal samples were collected by placing rats in clean, empty cages. The samples were then immediately transferred into cryovials, snap-frozen in liquid nitrogen, and stored at –80°C to preserve microbial integrity. Total genomic DNA was extracted from the samples using a commercial microbial DNA kit, with the resulting DNA serving as the template for high-throughput 16S rRNA gene sequencing. Purified amplicons were pooled in equimolar amounts and subjected to paired-end sequencing on an Illumina MiSeq PE300 platform or NovaSeq PE250 platform (Illumina, San Diego, USA) following standard protocols provided by Hangzhou LC-Bio Pharm Technology Co., Ltd. (Hangzhou, China). Bioinformatics analysis of the gut microbiota was conducted using the LC-Bio Cloud Platform (https://www.omicstudio.cn). The percentage of variation explained by the treatment and its statistical significance were assessed using the PERMANOVA test as implemented in the Vegan v2.5-3 package. To identify significantly abundant bacterial taxa among different groups, the linear discriminant analysis (LDA) effect size was evaluated using the LEfSe online tool.

**Metabolomics Analysis**

Metabolites from fecal samples were extracted for untargeted metabolomic profiling. Briefly, freshly collected fecal samples were weighed, washed with PBS to remove impurities, and homogenized. The homogenates were centrifuged, and the resulting pellets were immediately frozen in liquid nitrogen for 1 h and then stored at −80°C until further use. For metabolite extraction, samples were slowly thawed at 4°C and resuspended in pre-cooled methanol/acetonitrile/water (2:2:1, v/v/v) containing deuterated internal standards. Three freeze-thaw cycles (liquid nitrogen freezing for 1 min followed by room temperature thawing) were applied to promote metabolite release. Samples were then sonicated for 10 min at 4°C and subjected to protein precipitation at −40°C. After centrifugation, the supernatant was collected for LC-MS/MS analysis. Untargeted metabolomics was performed using a UHPLC system (Vanquish, Thermo Fisher Scientific) equipped with a Waters ACQUITY UPLC BEH Amide column (2.1 mm × 50 mm, 1.7 μm) coupled to an Orbitrap Exploris 120 mass spectrometer (Thermo). Raw data were converted into mzXML format and subsequently processed and analyzed using R software (version 4.0.0).

**References**

[1] Prut L, Belzung C. The open field as a paradigm to measure the effects of drugs on anxiety-like behaviors: a review. Eur J Pharmacol 2003;463:3–33. https://doi.org/10.1016/s0014-2999(03)01272-x.

[2] Longa EZ, Weinstein PR, Carlson S, Cummins R. Reversible middle cerebral artery occlusion without craniectomy in rats. Stroke 1989;20:84–91. https://doi.org/10.1161/01.STR.20.1.84.
