# Supplementary figures and images for: Sleep Deprivation Exacerbates Ischemic Stroke Outcomes via Akkermansia Depletion and Metabolic Dysregulation
Source: CNS Neurosci Ther. 2026 May 20;32(5):e70933. doi: 10.1002/cns.70933 (PMC13240125; doi:10.1002/cns.70933)

**Figure S1**. Overview of animal treatments and experimental design.

**
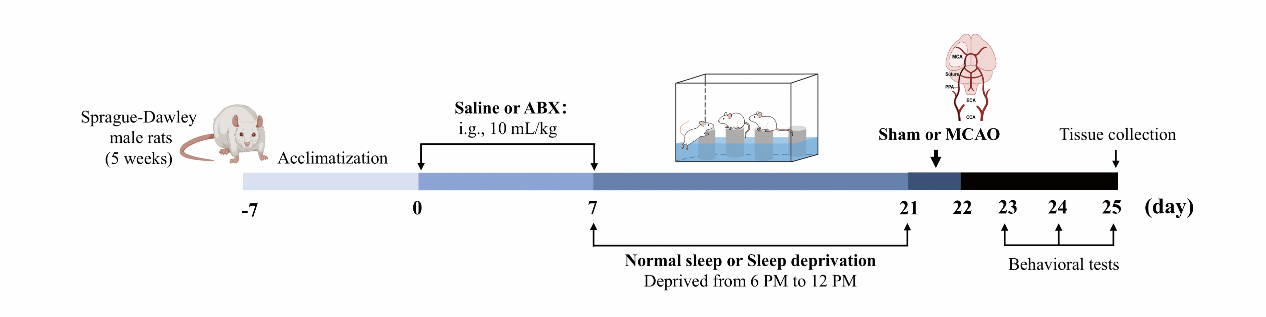
**

Supplement: Supplementary file 2 — Figure S1: Overview of animal treatments and experimental design. [file CNS-32-e70933-s005.docx]
