# Supplementary material for: Sleep Deprivation Exacerbates Ischemic Stroke Outcomes via Akkermansia Depletion and Metabolic Dysregulation
Source: CNS Neurosci Ther. 2026 May 20;32(5):e70933. doi: 10.1002/cns.70933 (PMC13240125; doi:10.1002/cns.70933)
Supplement: Supplementary file 3 — Figure S2: SD potentiates central and peripheral pro‐inflammatory responses in ABX‐treated rats. (A–C) Quantitative analysis of IL‐1β (A), IL‐6 (B), and TNF‐α (C) protein levels in the cerebral cortex by ELISA (n = 6). (D–F) Quantitative analysis of IL‐1β (D), IL‐6 (E), and TNF‐α (F) protein levels in serum by ELISA (n = 6). Significance levels: *p < 0.05, **p < 0.01, and ***p < 0.001. [file CNS-32-e70933-s003.docx]

**Figure S2** SD potentiates central and peripheral pro-inflammatory responses in ABX-treated rats. (A-C) Quantitative analysis of IL-1β (A), IL-6 (B), and TNF-α (C) protein levels in the cerebral cortex by ELISA (n = 6). (D-F) Quantitative analysis of IL-1β (D), IL-6 (E), and TNF-α (F) protein levels in serum by ELISA (n = 6). Significance levels: **p* < 0.05, ** *p* < 0.01, and *** *p* < 0.001.

**
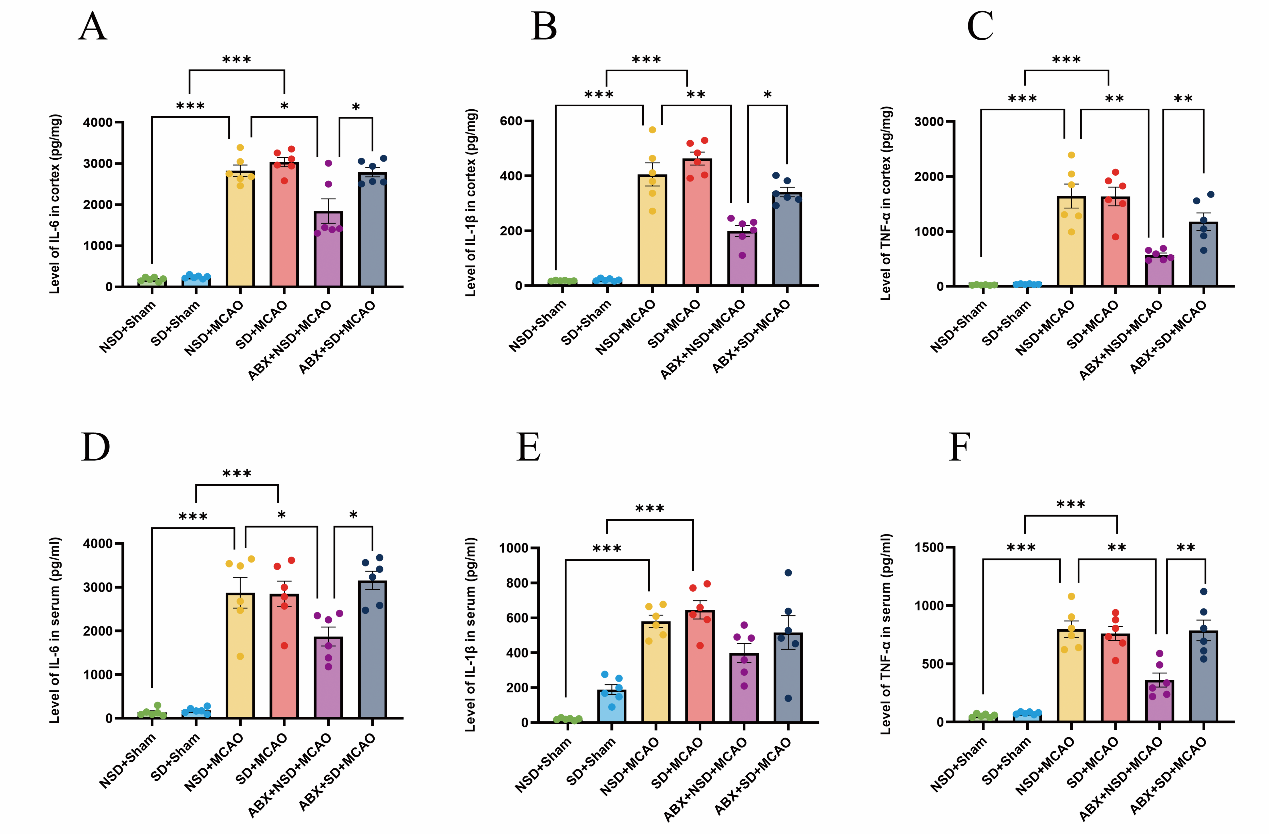
**
