# Supplementary material for: Sleep Deprivation Exacerbates Ischemic Stroke Outcomes via Akkermansia Depletion and Metabolic Dysregulation
Source: CNS Neurosci Ther. 2026 May 20;32(5):e70933. doi: 10.1002/cns.70933 (PMC13240125; doi:10.1002/cns.70933)
Supplement: Supplementary file 4 — Figure S3: ABX treatment significantly alters gut microbial diversity and composition after stroke, but its regulatory effect is attenuated under SD. (A) PCoA based on Bray‐Curtis distances. (B, C) Alpha diversity indices of the gut microbiota, including the Chao1 and Shannon indices. (D) Relative abundances of microbial taxa at the phylum level across experimental groups. (E–J) Comparative analyses of the relative abundances of major bacterial phyla: Bacteroidota, Firmicutes, Verrucomicrobiota, Proteobacteria, Actinobacteriota, and Desulfobacterota among different experimental groups. Significance levels: *p < 0.05, **p < 0.01, and ***p < 0.001. [file CNS-32-e70933-s006.docx]

**Figure S3** ABX treatment significantly alters gut microbial diversity and composition after stroke, but its regulatory effect is attenuated under SD. (A) PCoA based on Bray-Curtis distances. (B-C) Alpha diversity indices of the gut microbiota, including the Chao1 and Shannon indices. (D) Relative abundances of microbial taxa at the phylum level across experimental groups. (E-J) Comparative analyses of the relative abundances of major bacterial phyla: Bacteroidota, Firmicutes, Verrucomicrobiota, Proteobacteria, Actinobacteriota, and Desulfobacterota among different experimental groups. Significance levels: * *p* < 0.05, ** *p* < 0.01, and *** *p* < 0.001.

**
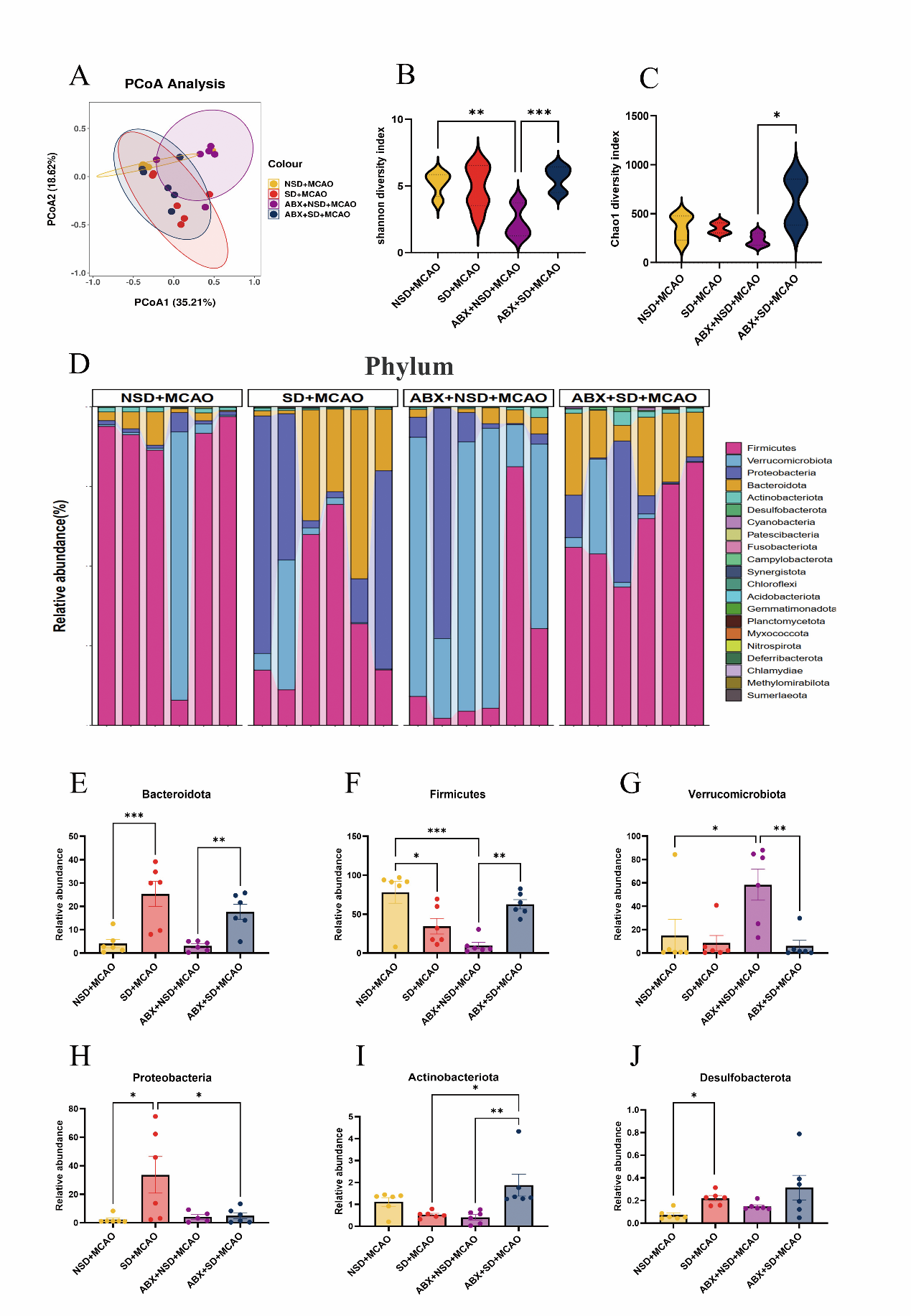
**
