# Supplementary material for: Sleep Deprivation Exacerbates Ischemic Stroke Outcomes via Akkermansia Depletion and Metabolic Dysregulation
Source: CNS Neurosci Ther. 2026 May 20;32(5):e70933. doi: 10.1002/cns.70933 (PMC13240125; doi:10.1002/cns.70933)
Supplement: Supplementary file 5 — Table S1: Baseline characteristics of the patients. [file CNS-32-e70933-s004.docx]

**Table S1.** Baseline characteristics of the patients.

| Variable | Non-insomnia (n=186) | Insomnia (n=114) | p-value |
| --- | --- | --- | --- |
| Age, mean ± SD (years) | 65.8 ± 11.9 | 65.7 ± 14.3 | 0.753 |
| Sex (Male), n (%) | 125 (67.2%) | 86 (75.4%) | 0.166 |
| Hypertension, n (%) | 131 (70.4%) | 81 (71.1%) | >0.99 |
| Diabetes mellitus, n (%) | 47 (25.3%) | 32 (28.1%) | 0.689 |
| Previous stroke, n (%) | 28 (15.1%) | 16 (14%) | 0.941 |
| Atrial fibrillation, n (%) | 64 (34.4%) | 42 (36.8%) | 0.761 |
| Coronary artery disease, n (%) | 22 (11.8%) | 10 (8.8%) | 0.522 |
| NIHSS score, median (IQR) | 15 (11–19) | 17 (13–20.8) | 0.026 |
| ASPECTS score, median (IQR) | 10 (9–10) | 10 (9–10) | 0.437 |
| Onset-to-reperfusion time (min), median (IQR) | 240 (180–360) | 240 (180–360) | 0.562 |
| Puncture-to-reperfusion time (min), median (IQR) | 110 (85–148.8) | 120 (90–150) | 0.297 |
| Intravenous thrombolysis, n (%) | 71 (38.2%) | 38 (33.3%) | 0.470 |
| Successful reperfusion (mTICI 2b-3), n (%) | 166 (89.2%) | 103 (90.4%) | 0.913 |

Note: IQR = interquartile range; SD = standard deviation. A *p-value* < 0.05 was considered statistically significant.
