# Supplementary material for: Sleep Deprivation Exacerbates Ischemic Stroke Outcomes via Akkermansia Depletion and Metabolic Dysregulation
Source: CNS Neurosci Ther. 2026 May 20;32(5):e70933. doi: 10.1002/cns.70933 (PMC13240125; doi:10.1002/cns.70933)
Supplement: Supplementary file 6 — Table S2: Comparison of study endpoints. [file CNS-32-e70933-s001.docx]

**Table S2.** Comparison of study endpoints.

| Outcomes | Non-insomnia (n=186) | Insomnia (n=114) | Adjusted value**^†^** (95% CI) | p-value |
| --- | --- | --- | --- | --- |
| Clinical outcomes |  |  |  |  |
| Immediate mRS score, median (IQR) | 5 (4–5) | 5 (4–5) | MD: 0.1 (-0.1–0.3) | 0.220 |
| 48-hour mRS score, median (IQR) | 4 (4–5) | 5 (4–5) | MD: 0.1 (-0.1–0.4) | 0.060 |
| 7-day mRS score, median (IQR) | 4 (3–5) | 5 (3.2–5) | MD: 0.2 (-0.2–0.5) | 0.007 |
| 90-day mRS score, median (IQR) | 3 (1–4) | 4 (2–4.8) | MD: 0.3 (-0.1–0.7) | 0.029 |
| Poor functional outcome at 90 days (mRS 3–6), n (%) | 107 (57.5%) | 77 (67.5%) | OR: 1.22 (0.66–2.24) | 0.089 |
| Severe complications and mortality |  |  |  |  |
| 90-day mortality, n (%) | 29 (25.9%) | 22 (32.4%) | OR: 0.96 (0.43–2.13) | 0.395 |
| 90-day stroke recurrence, n (%) | 19 (17%) | 21 (30.9%) | OR: 2.72 (1.19–6.37) | 0.038 |
| Intracerebral hemorrhage, n (%) | 75 (40.3%) | 53 (46.5%) | OR: 0.94 (0.54–1.65) | 0.336 |
| Symptomatic intracranial hemorrhage, n (%) | 35 (18.8%) | 15 (13.2%) | OR: 0.54 (0.25–1.14) | 0.264 |
| In-hospital pneumonia, n (%) | 111 (59.7%) | 71 (62.3%) | OR: 0.70 (0.39–1.25) | 0.715 |
| Laboratory parameters |  |  |  |  |
| Fibrinogen (g/L), median (IQR) | 2.7 (2.2–3.2) | 2.7 (2.1–3.2) | MD: -0 (-0.3–0.2) | 0.980 |
| D-dimer (mg/L), median (IQR) | 1.0 (0.5–2.9) | 1.2 (0.6–2.9) | MD: 0.09 (-0.15–0.35) | 0.474 |
| White blood cell count (×10⁹/L), median (IQR) | 9.3 (7.6–10.9) | 9.5 (7.9–13.1) | MD: 1.7 (0.9–2.5) | 0.022 |
| Neutrophil percentage (%), median (IQR) | 83.2 (74.4–89.6) | 85.1 (78.5–90.3) | MD: 6.9 (2.3–11.4) | 0.032 |

Note: Data are presented as median (IQR) or mean ± SD, as appropriate. OR = odds ratio (95% confidence interval); MD = mean difference (95% confidence interval).

**^†^** Adjusted ORs (aOR) and adjusted mean differences (aMD) were obtained using logistic regression or linear regression models, respectively, with adjustment for age, sex, baseline NIHSS score, baseline ASPECTS, hypertension, diabetes mellitus, atrial fibrillation, intravenous thrombolysis, and onset-to-reperfusion time. A *p-value* < 0.05 was considered statistically significant.
